# Curcumin Synergistically Enhances the Cytotoxicity of Arsenic Trioxide in U266 Cells by Increasing Arsenic Uptake

**DOI:** 10.1155/2021/3083041

**Published:** 2021-10-12

**Authors:** Dingding Han, Guibo Ma, Yujuan Gao, Yanhua Su

**Affiliations:** Hematology Department, The First Affiliated Hospital of Harbin Medical University, Harbin 150001, China

## Abstract

Despite the constant emergence of new methods for the treatment of multiple myeloma (MM), relapse and drug resistance still exist, especially in MM with p53 mutations. Arsenic trioxide (ATO) can be used in MM treatment, but this single drug has poor effectiveness and also side effects. Curcumin is a safe and effective compound that can enhance the anticancer effects of many drugs. Previous studies have suggested that tumor cell sensitivity to ATO is related to the intracellular arsenic content, and aquaporin 9 (AQP9) is the key factor that determines intracellular arsenic content. This study aimed to explore whether curcumin can increase ATO cytotoxicity in MM and whether the mechanism is related to the regulation of intracellular arsenic content. U266 was treated with ATO, curcumin, and their combination, and cell proliferation, apoptosis, and intracellular arsenic content were detected by CCK-8 assay, flow cytometry, and HPLC-ICP-MS, respectively. AQP9 mRNA and protein levels were detected by qPCR and western blotting. The levels of Mcl-1, Bcl-2, Bax, caspase-3, and cleaved caspase-3 protein were detected by western blotting. ATO-induced cytotoxicity to U266 occurred in a time- and dose-dependent manner, but the therapeutic efficacy at low drug concentrations was modest. The arsenic content in U266 was lower than that in NB4, and the arsenic uptake by U266 was concentration-dependent. The expression levels of AQP9 mRNA and AQP9 protein in U266 were lower than those in NB4. Curcumin significantly enhanced the lethality of ATO to U266. The arsenic content in U266 in the combined drug group increased significantly compared with ATO treatment alone. After curcumin treatment, the AQP9 mRNA and AQP9 protein expression levels in U266 also increased. Compared with the control group, the expression of antiapoptotic proteins Mcl-1 and Bcl-2 decreased, the expression of proapoptotic protein Bax increased, the ratio of Bax/Bcl-2 increased, and the expression of caspase-3 decreased and cleaved caspase-3 increased in the combined drug groups. Curcumin can enhance the killing effects of ATO on U266 by increasing the intracellular arsenic content, which may be related to the upregulation of AQP9 expression. The combination of these two drugs is expected to be a potential clinical treatment for MM.

## 1. Introduction

Multiple myeloma (MM) is the second most common hematological malignancy, accounting for 13% of all cases [1–3]. MM is characterized by the abnormal, malignant proliferation of clonal plasma cells in the bone marrow. Although there are many new methods to treat MM, such as drugs, hematopoietic stem cell transplantation (HSCT), chimeric antigen receptor T-cell immunotherapy (CAR-T), monoclonal antibodies, and other immunotherapies, it is still an incurable disease, and the majority of patients easily relapse and develop chemoresistance. Additionally, the treatment of patients with p53 mutations, abnormal karyotypes, relapse, and refractoriness is more difficult. Although current treatment methods extend the overall survival rates of patients with MM, side effects limit many patients from choosing more effective treatment methods. Therefore, this study expects to use the advantages of traditional Chinese medicine to seek more effective treatment methods to improve patient outcomes.

ATO is the first-line drug for acute promyelocytic leukemia (APL). In addition, many studies have shown that ATO can be used to treat MM; the sensitivity of MM to ATO is concentration-dependent, the inhibitory effects of low concentrations are weak, and increasing the concentration of ATO will increase its inhibitory effect [4]. It is feasible to administer only ATO to MM patients, but the curative effects are limited, and the toxicity is significant. Further research is needed to improve the biodistribution and efficacy of drugs. Combined chemotherapy may improve the effects of ATO and reduce its toxicity [5]. Curcumin is a safe, economical, and effective molecule that has been shown to inhibit the proliferation of cancer cells while simultaneously modulating multiple cell signaling pathways to reduce or prevent many different types of cancers [6]. Many studies have found that curcumin can overcome resistance to dexamethasone, doxorubicin, and other drugs and enhance the effects of bortezomib, thalidomide, and other drugs without increasing the side effects [7, 8]. In addition, curcumin can inhibit MM cell lines with abnormal karyotypes, such as del (17p), *t* (11; 14), *t* (4; 14), and *t* (14; 16), and primary MM cells are also sensitive to curcumin [9]. However, whether curcumin and ATO have synergistic effects has not been determined. To date, there has been no research on curcumin combined with ATO in the treatment of MM.

According to literature reviews, arsenic needs to enter cells before playing a cytotoxic role, and the intracellular arsenic content determines the arsenic cytotoxicity. One reason for the poor sensitivity of cells to arsenic is the low intracellular arsenic content [10–12]. The process of arsenic uptake is the key factor that determines the intracellular arsenic content. AQP9 is an important membrane protein that plays a role as a channel to transfer water and various small solutes [13]. Many studies have indicated that AQP9 is the main protein responsible for arsenic entry into cells [11, 12, 14, 15], and its expression level affects the intracellular arsenic content. Therefore, we propose that the poor effects of ATO on MM may be related to the intracellular arsenic content.

To determine whether curcumin could potentiate the sensitivity of multiple myeloma cells to ATO and to specify the mechanism by which this happens, the present study investigated how curcumin affected AQP9 and intracellular arsenic content in human MM cell line U266 with p53 mutations. This study first found that curcumin enhanced U266 cell inhibition after ATO treatment by increasing the intracellular arsenic content, which may be related to the upregulation of AQP9 expression. Curcumin combined with ATO is expected to become a clinical treatment for MM. Curcumin can not only increase ATO inhibition of MM but also reduce the dosage of ATO needed to reduce its toxicity, increase its efficiency, and expand the application scope of ATO in clinical treatment.

## 2. Materials and Methods

### 2.1. Materials and Reagents

The human MM cell line U266 was purchased from the Institute of Basic Medicine, Chinese Academy of Medical Sciences. The human APL cell line NB4 was obtained from the Blood Center Laboratory of the First Affiliated Hospital of Harbin Medical University. RPMI-1640 and fetal bovine serum (FBS) were obtained from Gibco (Grand Island, NY, USA). ATO was purchased from Medical University Pharmaceutical Industry (Harbin, Heilongjiang). Curcumin was purchased from Sigma-Aldrich (St. Louis, MO, USA). The anti-AQP9 antibody was purchased from Santa Cruz Biotechnology (Santa Cruz, CA, USA). Mcl-1, Bcl-2, and Bax antibodies were purchased from Abcam (Cambridge, MA, USA). Caspase-3/cleaved caspase-3 antibodies were purchased from Cell Signaling Technology (Danvers, MA, USA).

### 2.2. Experimental Methods

#### 2.2.1. Cell Culture

U266 and NB4 cells were cultured in RPMI-1640 with 10% FBS, 100 U/ml penicillin, and 100 *µ*g/ml streptomycin, at 37°C in a humidified atmosphere with 5% CO_2_, and the medium was changed approximately every 2-3 days.

#### 2.2.2. Assessment of Apoptosis by Flow Cytometry

Cells from different treatment groups were taken and washed twice with precooled binding buffer. The cells were then suspended in 100 *μ*l of binding buffer solution. Then, 5 *μ*l of annexin V-FITC and 10 *μ*l of PI were added to the suspended cells followed by incubation at room temperature (20–25°C) in the dark for 15 min. Then, the binding buffer was added to a total volume of 500 *μ*l. The apoptosis level was detected by flow cytometry (BD Biosciences, San Jose, CA, USA).

#### 2.2.3. Cell Proliferation Assay by CCK-8 Kit

A 100 *μ*l cell suspension from each treatment group was incubated in 96-well plates in a humidified atmosphere with 5% CO_2_ for 24 h. Then, 10 *μ*l of CCK-8 reagent (Dojindo Laboratories, Tokyo, Japan) was added to each well for 4 h. Finally, the absorbance of each well was measured at 450 nm with a microplate reader.

#### 2.2.4. Intracellular Arsenic Content by HPLC-ICP-MS

Cells from each treatment group were washed with PBS three times and then suspended in PBS. The supernatant was discarded after cell counting, and the cell precipitate was used for the intracellular arsenic assay. Cell samples from each treatment group were removed, 49 *μ*l of mobile phase was added for dilution, and then 1 *μ*l of 5% ammonia was added followed by mixing well to obtain the treatment solution. Then, 50 *μ*l of 30% hydrogen peroxide was added to the above treatment solution followed by vortexing to mix well with standing for 20 min to obtain the pretreatment sample. Next, 90 *μ*l of the pretreatment sample was added to 20% perchloric acid for 10 min of incubation. After centrifugation at 4°C and 13200 rpm for 15 min, the supernatant was filtered through a 0.22 *μ*M filter membrane to obtain the test sample, which was examined on high-performance liquid chromatography (HPLC, Altus, PerkinElmer, USA) in combination with inductively coupled plasma mass spectrometry (ICP-MS, NexION 350D, PerkinElmer, USA).

#### 2.2.5. Western Blotting

Cells from different treatment groups were collected and mixed with lysis solution on ice for 30 min. The mixture was centrifuged at high speed and low temperature (4°C, 12000 rpm) for 15 min, and the protein concentration was determined by the BCA method. The same amount of protein was transferred by SDS-PAGE to a PVDF membrane. The membrane was blocked with a 5% skimmed milk powder sealing solution, and the PVDF membrane was incubated with the primary antibody at 4°C overnight. *β*-Actin, *β*-tubulin, and GAPDH were used as loading positive. After washing with TBST three times, the secondary antibody was added followed by incubation for 1 h. The ECL color was developed and detected on the computer and the strip was analyzed semiquantitatively by Image Lab software.

#### 2.2.6. RNA Extraction and Quantitative Real-Time PCR (qPCR)

Total RNA was isolated using TRIzol reagent (Invitrogen, Carlsbad, CA, USA) according to the manufacturer's instructions. The RNA concentration was measured by absorbance of ultraviolet light at a wavelength of 260/280 nm, using the BioPhotometer (Hamburg, Germany). We performed reverse transcription using Universal RT-PCR Kit (Solarbio, Beijing, China) to obtain cDNA templates. qPCR was performed using SYBR Green Fast qPCR Mix (ABclonal, Beijing, China), according to the manufacturer's instructions. qPCR was conducted using SYBR solution (5 ul), primer (0.6 ul), and template DNA (4.4 *µ*l). The reaction conditions for qPCR were 95°C for 5 min, followed by 40 cycles of 95°C for 10 sec, 60˚C for 30 sec, and 68°C for 50 sec. Primers for AQP9 and *β*-actin were obtained from Sangon Biotech (Shanghai, China). *β*-Actin was used as an internal control. Primer sequences for the analyzed genes are shown in Table 1.

#### 2.2.7. Combination Index (CI)

The CI was analyzed by CompuSyn software. This software is based on the multiple drug effect equation set by Chou TC and Talalay P [16]. CI < 1 was defined as a synergistic effect, CI > 1 was defined as antagonism, and CI = 1 was defined as an additive effect.

#### 2.2.8. Statistical Analysis

A *t*-test was used for analysis between two groups, and one-way ANOVA and Tukey's multiple comparison tests were for analysis used between multiple groups. All values are expressed as the mean ± standard deviation (SD), and the difference was statistically significant when *P* < 0.05. All analyses were completed by GraphPad Prism 7.0 software. All experiments were repeated three times.

## 3. Results

### 3.1. ATO Inhibited U266 Cells in a Time- and Dose-Dependent Manner

**Figure 1:**
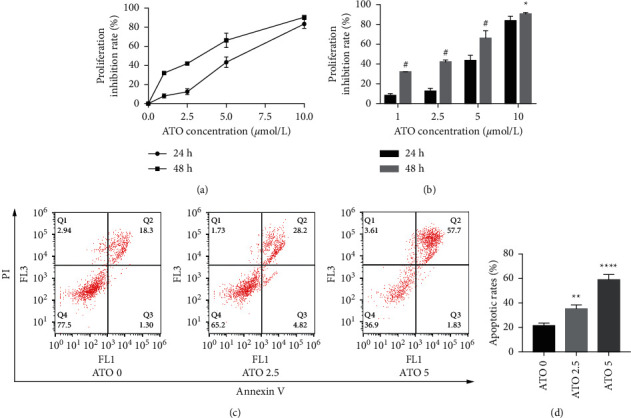
Inhibitory effects of ATO on U266 cells. (a, b) The inhibition rates of U266 cells treated with different concentrations of ATO (0, 1, 2.5, 5, and 10 *μ*mol/L) for 24/48 h. ^*∗*^ indicates *P* < 0.05 for ATO treatment after 48 h in U266 cells compared with 24 h and # indicates *P* < 0.0001. (c, d) The apoptosis rates of U266 cells treated with different concentrations of ATO (0, 2.5, and 5 *μ*mol/L) for 48 h. ^*∗∗*^ indicates *P* < 0.01 for the ATO 2.5 treatment group compared with the untreated group; ^*∗∗∗∗*^ indicates *P* < 0.0001 for the ATO 5 treatment group compared with the untreated group.

As shown in Figure 1(a), with increasing ATO concentration, the inhibitory effects of ATO on U266 cells gradually increased, and the difference was statistically significant (*F* = 228.3/138.1 for U266 24 h/48 h, *P* < 0.0001). Multiple *t*-test analysis showed that the longer the treatment time was, the more obvious the inhibitory effect was (Figure 1(b)). However, the inhibition rates of U266 cells treated with low ATO concentrations (1 and 2.5 *μ*mol/L) for 24/48 h were (8.05 ± 3.25)/(32.04 ± 3.25)% and (12.52 ± 3.25)/(42.05 ± 3.25)%, respectively, which are both less than 50%. The IC50 for 24/48 h was 5.45/4.99 *μ*mol/L. As shown in Figures 1(c) and 1(d), with increasing ATO concentration, the apoptosis rate of U266 cells increased gradually, and the result was significantly different by one-way ANOVA (*F* = 88, *P* < 0.0001). However, the apoptosis rate of U266 cells treated with 2.5 *μ*mol/L ATO for 48 h was only 35.10 ± 2.84%. Therefore, this study suggested that ATO inhibited the proliferation of U266 cells in a time- and dose-dependent manner, but the therapeutic efficacy of low drug concentrations was modest.

### 3.2. The Arsenic Content in U266 Cells Was Lower Than That in NB4 Cells, and the Arsenic Uptake Was Concentration-Dependent

**Figure 2:**
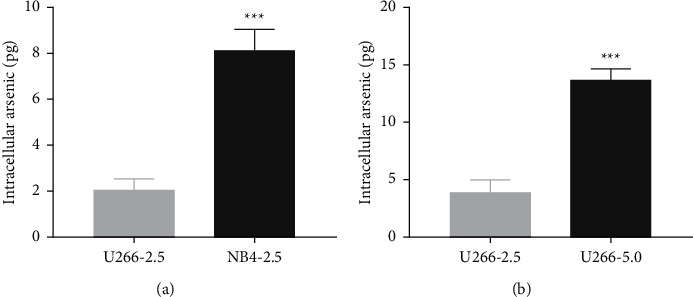
Arsenic content in U266 and NB4 cells treated with ATO (48 h). (a) The U266 and NB4 cells treated with ATO (2.5 *μ*mol/L) for 48 h. (b) The U266 cells treated with different concentrations of ATO (2.5 and 5 *μ*mol/L) for 48 h; ^*∗∗∗*^ indicates *P* < 0.001.

To study whether the sensitivity of U266 cells to ATO is related to the intracellular arsenic content, the APL cell line NB4 was used as the positive control, and the intracellular arsenic content was detected by HPLC-ICP-MS. As shown in Figure 2(a), U266 and NB4 cells were treated with 2.5 *μ*mol/L ATO for 48 h, the arsenic content in U266 cells was lower than that in NB4 cells, and the difference was statistically significant (*t* = 9.627, *P*=0.0007). As shown in Figure 2(b), with increasing ATO concentration, the arsenic content in U266 cells increased. Arsenic uptake by U266 cells was concentration-dependent (*t* = 10.48, *P*=0.0005).

### 3.3. Curcumin Enhanced ATO-Induced Cytotoxicity in U266 Cells and Increased Intracellular Arsenic Content

**Figure 3:**
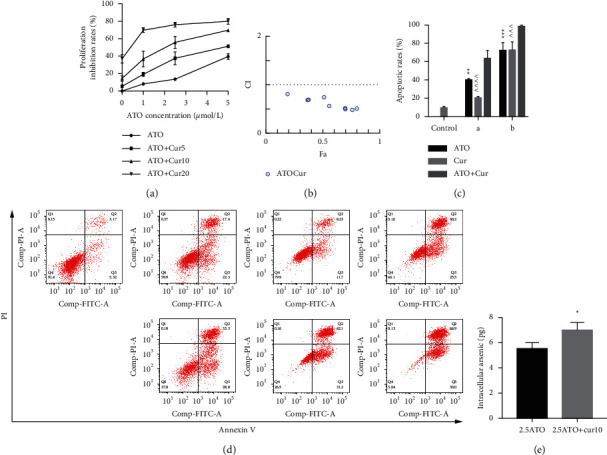
Analysis of U266 cells treated with ATO, curcumin, and their combination. (a) The inhibition rates. (b) The analysis of the curcumin and ATO combined application index. (c, d) The apoptotic rates. ^*∗∗*^ indicates *P* < 0.01 for the combination group compared with the ATO alone groups; ^*∗∗∗*^ indicates *P* < 0.001; ^∧∧∧^ indicates *P* < 0.001 for the combination group compared with curcumin alone; and ^∧∧∧∧^ indicates *P* < 0.0001. Group a: ATO 2.5 *μ*mol/L, curcumin 10 *μ*mol/L; group b: ATO 5 *μ*mol/L, curcumin 20 *μ*mol/L. (e) The intracellular arsenic content. ^*∗*^ indicates *P* < 0.05 for the combination group compared with ATO treatment alone.

To study the synergistic effects between ATO and curcumin, U266 cells were treated with each drug alone or their combination, and proliferation, apoptosis, and intracellular arsenic content were detected. As shown in Figure 3(a), compared with the application of ATO or curcumin alone, the combination of curcumin and ATO increased the inhibition rate of the cells. Additionally, with increasing curcumin concentration, the inhibition rate was more obvious with a significant difference. As shown in Figure 3(b) and Table 2, combination index analysis demonstrated that ATO and curcumin synergistically inhibited the proliferation of U266 cells. As shown in Figures 3(c) and 3(d), compared with the single drug groups, the combination of drugs increased cell apoptosis. As the concentrations increased, apoptosis became more obvious, and the difference was statistically significant. As shown in Figure 3(e), the arsenic content in U266 cells treated with ATO combined with curcumin was higher than that in the ATO alone group (*t* = 3.3226, *P*=0.0321). Therefore, our study suggests that curcumin increased the inhibitory effects of ATO on U266 cells by increasing the arsenic content.

### 3.4. The Expression Level of AQP9 in U266 Cells Was Lower Than That in NB4 Cells, and Curcumin Upregulated U266 AQP9 Expression

**Figure 4:**
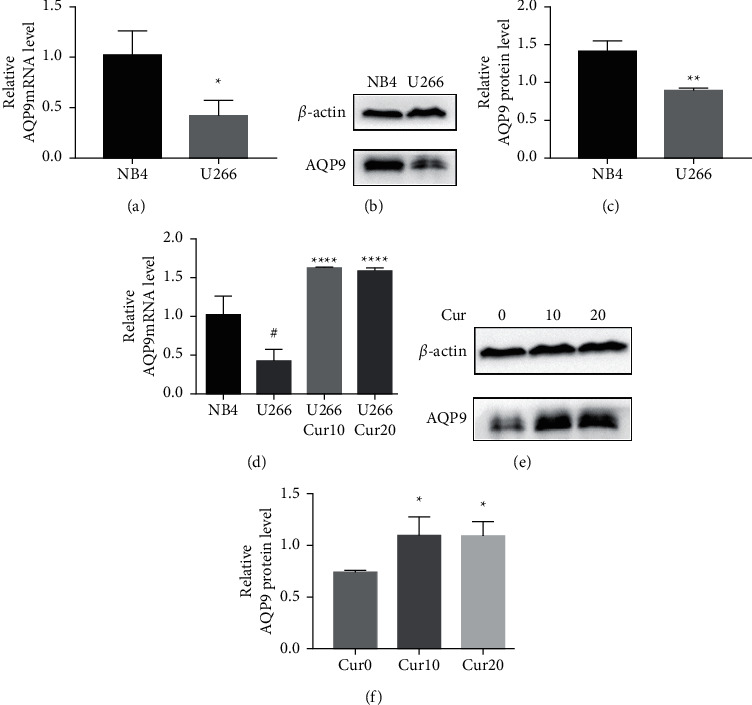
Analysis of AQP9 in U266 and NB4 cells. (a–c) The differences in AQP9 mRNA and AQP9 protein levels in U266 and NB4 cells. ^*∗*^ indicates *P* < 0.05 for U266 cells compared with NB4 cells; ^*∗∗*^ indicates *P* < 0.01. (c–e) The effects of curcumin on the expression of AQP9 mRNA and AQP9 protein levels in U266 cells. # indicates *P* < 0.01 for U266 cells compared with NB4 cells. ^*∗*^ indicates *P* < 0.05 for U266 cells with curcumin treatment compared with the control group. ^*∗∗∗∗*^ indicates *P* < 0.0001.

As shown in Figures 4(a)–4(c), the AQP9 mRNA and AQP9 protein levels in U266 cells were lower than those of NB4 cells, and the difference was statistically significant (*t* = 3.567, *P*=0.0234 and *t* = 6.23, *P*=0.0034). As shown in Figure 4(d), the expression of AQP9 mRNA significantly increased compared with that in the control group (*F* = 43.98, *P* < 0.0001). As shown in Figures 4(e) and 4(f), after curcumin treatment of U266 cells, the expression level of the AQP9 protein also increased, and the result was significantly different by one-way ANOVA (*F* = 6.556, *P*=0.0309). Therefore, our study suggests that the low sensitivity of U266 cells to ATO is related to the low intracellular arsenic content, which may be due to the low expression levels of AQP9 mRNA and the AQP9 protein in U266 cells, which leads to an insufficient arsenic uptake capacity by U266 cells. Curcumin can increase the inhibitory effects of ATO on U266 cells, which may be related to its regulation of U266 cell AQP9 mRNA and protein expression, thus increasing arsenic uptake and increasing the sensitivity of U266 cells to ATO.

### 3.5. Effects of ATO and Curcumin on Apoptosis-Related Proteins in U266 Cells

**Figure 5:**
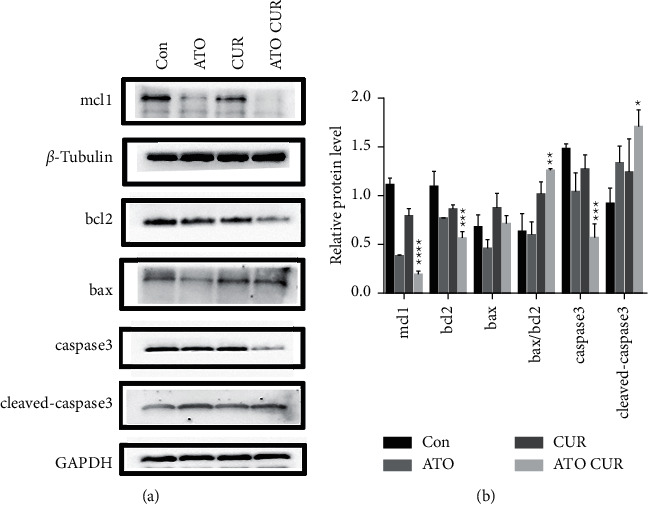
Effects of ATO and curcumin on the levels of cell apoptosis-related proteins. ^*∗*^ indicates *P* < 0.05 for the combination group compared with control; ^*∗∗*^ indicates *P* < 0.01; ^*∗∗∗*^ indicates *P* < 0.001; ^*∗∗∗∗*^ indicates *P* < 0.0001. U266 treated with ATO 2.5 *μ*mol/L, curcumin 10 *μ*mol/L, and their combination for 48 h.

As shown in Figure 5, compared with the control group, the expression of antiapoptotic proteins Mcl-1 and Bcl-2 decreased, while the expression of proapoptotic protein Bax increased, the ratio of Bax/Bcl-2 increased, the expression of caspase-3 decreased, and the expression of cleaved caspase-3 increased in the combined treatment group; the difference was statistically significant.

## 4. Discussion

We found that ATO inhibited the proliferation and promoted apoptosis of U266 cells in a time- and dose-dependent manner. However, the inhibition rates of U266 cells treated with low ATO concentrations (1 and 2.5 *μ*mol/L) for 24/48 h were (8.05 ± 3.25)/(32.04 ± 3.25)% and (12.52 ± 3.25)/(42.05 ± 3.25)%, respectively, which are less than 50%. The IC50 for 24/48 h was 5.45/4.99 *μ*mol/L. The apoptosis rate of U266 cells treated with 2.5 *μ*mol/L ATO for 48 h was only 35.10 ± 2.84%. Here, we see that the therapeutic efficacy of low drug concentrations is modest, which is consistent with the fact that the therapeutic effects of ATO on MM are not as good as those of APL. Therefore, what causes different diseases to have different sensitivities to arsenic? The uptake of drugs is usually a necessary process for the drugs to play their role. Bhattacharjee et al. [10] found that the entry of arsenic into cells is the key step for it to cause cytotoxicity; enhancing the ability of arsenic to be taken up by cells can increase cell sensitivity to ATO. To study whether the sensitivity of U266 cells to ATO is related to the intracellular arsenic content, we conducted relevant experiments and concluded that, compared with NB4 cells, the arsenic content in U266 cells was much lower than that in NB4 cells, and the difference was statistically significant. With increasing ATO concentration, the arsenic content in U266 cells increased. The above results showed that the cytotoxic effects of low ATO concentrations on U266 cells were lower, and with increasing ATO concentration, the inhibition rate and apoptosis rate of U266 cells increased. We considered that the reason for the low sensitivity of U266 cells to arsenic might be their low intracellular arsenic content. With increasing ATO concentration, the intracellular arsenic content would increase, enhancing the cytotoxic effects of ATO. Leung et al. [11] also found that K562 cells had low sensitivity to ATO, when they increased arsenic uptake, the sensitivity of K562 cells to ATO increased. Chau et al. [12] treated different types of leukemia cell lines with ATO and ATO + azacytidine and determined intracellular arsenic concentrations and apoptosis rates; it was concluded that the intracellular arsenic content in the combined treatment group was higher than that in the group treated with ATO alone, and combined treatment led to an increase in apoptosis rate, indicating that azacytidine combined with ATO plays a cytotoxic role by increasing arsenic uptake. Different cells have different sensitivities to arsenic, which depend on their intracellular arsenic content. The reason for the poor therapeutic effects of ATO against MM may be that the arsenic uptake ability of myeloma cells is insufficient because the intracellular arsenic content is not high enough to inhibit the proliferation of the tumor cells. Increasing the concentration of ATO can increase the intracellular arsenic content and then increase the cytotoxic effects of ATO, but the side effects will also increase, which limits the clinical applications of ATO.

Rousselot et al. [5] also showed that ATO alone has limited efficacy and significant toxicity in MM patients; further research is needed to improve the biological distribution and efficacy of this drug, and a combination of drugs may improve the effects of ATO and reduce its toxicity. As mentioned above, intracellular arsenic content is the key factor for arsenic to exert its cytotoxic effects. Previous studies confirmed that a combination of drugs that can increase intracellular arsenic content can enhance the sensitivity of tumor cells to arsenic. Therefore, we sought an appropriate drug combination to enhance the therapeutic effects of ATO on MM. Many studies have shown that curcumin can improve the effects of dexamethasone, bortezomib, and other antimyeloma drugs. However, there have been no reports of curcumin combined with ATO for the treatment of MM. Therefore, in this study, U266 cells were treated with ATO and curcumin alone and in combination to detect cell proliferation, cell apoptosis, and intracellular arsenic content. Our results showed that the inhibition and apoptosis rates of the curcumin combined with the ATO group were higher than those of the single drug groups. Combination index analysis demonstrated that the two drugs synergistically inhibit the proliferation of U266 cells. The intracellular arsenic content in U266 cells treated with curcumin combined with ATO was higher than that in the ATO single drug group. Our study showed for the first time that curcumin enhanced the inhibitory effects of ATO in U266 cells by increasing the intracellular arsenic content. In addition, our results showed that, compared with the control group, the expression of antiapoptotic proteins Mcl-1 and Bcl-2 decreased, the expression of proapoptotic protein Bax increased, the ratio of Bax/Bcl-2 increased, the expression of caspase-3 decreased, but the expression of cleaved caspase-3 increased in the combination group. Therefore, we think that curcumin combined with ATO inhibits U266 cells by regulating apoptosis-related proteins. Thus, curcumin combined with ATO is expected to be a potential treatment for MM. Curcumin can not only increase the inhibitory effects of ATO on MM but also lead to a reduction in the dosage of ATO needed to decrease toxicity, increase efficiency, and expand the application scope of ATO in clinical treatment.

Then, what is the mechanism of the difference in intracellular arsenic content? Arsenic uptake is the key factor in determining intracellular arsenic content. Aquaporins (AQPs), which have helical structures with six transmembrane helices arranged in the form of monomers, dimers, and tetramers, are a class of proteins that are widely distributed on biological cell membranes to form pores [17]. They are selective channels and are mainly involved in transport and various transport processes [18]. Currently, 13 kinds of AQPs (AQP0-12) have been found. AQP9 is an important membrane protein that plays a role as a channel for the transfer of water and various small solutes [13]. Leung et al. [11] found that HL-60 cells had a poor response to arsenic, the expression of AQP9 in HL-60 cells treated with all-trans retinoic acid (ATRA) + ATO was higher than that of HL-60 cells treated with ATO alone, the intracellular arsenic content also increased in these cells, and the cell survival rate significantly decreased. Chau et al. [12] conducted an experiment in which different types of leukemia cell lines were treated with ATO and ATO + azacytidine, and the intracellular arsenic, AQP9 expression, and apoptosis rates were detected; the results showed that intracellular arsenic contents and AQP9 expression in the combined treatment group were higher than those in the ATO alone group. The apoptosis rate also increased; this result indicated that azacytidine could increase the arsenic uptake of cells by upregulating the expression of AQP9 and then play a synergistic role with ATO. Herein, we observed that AQP9 is the key channel protein that allows arsenic to enter cells, and the expression level of AQP9 affects cellular arsenic uptake. To study whether the difference in arsenic content in U266 and NB4 cells is related to the expression level of AQP9, we detected the expression of AQP9 in both U266 and NB4 cells. This study found that the expression of AQP9 mRNA and the AQP9 protein in U266 cells was lower than that in NB4 cells, which was consistent with the lower expression of AQP9 in myeloma cell lines reported in [19]. From this, our study speculated that the lower arsenic content in U266 cells was due to the lower expression of AQP9 mRNA and the AQP9 protein. Further study showed that curcumin combined with ATO not only increased the intracellular arsenic content in U266 cells but also increased the expression of AQP9 mRNA and the AQP9 protein. Based on these results, we speculated that the mechanism by which curcumin increases arsenic content in U266 cells may be related to upregulation of the expression of AQP9 and enhancement of U266 cell arsenic uptake ability.

In summary, this study concluded that the poor effects of ATO in the treatment of MM may be related to intracellular arsenic content. We found, for the first time, that curcumin enhanced the inhibitory effects of ATO in U266 cells by increasing the intracellular arsenic content, which may be related to enhanced arsenic uptake. Curcumin combined with ATO is expected to become a potential treatment for MM and provide a new choice for relapsed and refractory patients.

## 5. Conclusions

Curcumin can enhance the killing effects of ATO on U266 by increasing the intracellular arsenic content, which may be related to the upregulation of AQP9 expression. The combination of these two drugs is expected to be a potential clinical treatment for MM.

## Figures and Tables

**Table 1:** Primer sequences for the analyzed genes.

Human *β*-actin-F	CCATCGTCCACCGCAAAT
Human *β*-actin-R	GCTGTCACCTTCACCGTTCC
Human AQP9-F	GAAGAGCAGCTTAGCGAAAGA
Human AQP9-R	ACAGCCACATCCAAGGACAAT

**Table 2:** Combinatorial effects of ATO and curcumin.

ATO (*μ*mol/L)	Cur (*μ*mol/L)	Combinatorial effect	CI
1.0	5.0	0.191	0.810
1.0	10.0	0.367	0.684
1.0	20.0	0.701	0.496
2.5	5.0	0.376	0.695
2.5	10.0	0.558	0.562
2.5	20.0	0.762	0.479
5.0	5.0	0.514	0.746
5.0	10.0	0.699	0.521
5.0	20.0	0.802	0.508

## Data Availability

The data used to support the findings of this study are available from the corresponding author upon request.

## References

[1] Wang S., Xu L., Feng J. (2019). Prevalence and incidence of multiple myeloma in urban area in China: a national population-based analysis. *Frontiers in oncology*.

[2] Palumbo A., Anderson K. (2011). Multiple myeloma. *New England Journal of Medicine*.

[3] Liu W., Liu J., Liu J. (2019). Mortality of lymphoma and myeloma in China, 2004–2017: an observational study. *Journal of Hematology & Oncology*.

[4] Liu Q., Hilsenbeck S., Gazitt Y. (2003). Arsenic trioxide-induced apoptosis in myeloma cells: p53-dependent G1 or G2/M cell cycle arrest, activation of caspase-8 or caspase-9, and synergy with APO2/TRAIL. *Blood*.

[5] Rousselot P., Larghero J., Arnulf B. (2004). A clinical and pharmacological study of arsenic trioxide in advanced multiple myeloma patients. *Leukemia*.

[6] Devassy J. G., Nwachukwu I. D., Jones P. J. H. (2015). Curcumin and cancer: barriers to obtaining a health claim. *Nutrition Reviews*.

[7] Sung B., Kunnumakkara A. B., Sethi G., Anand P., Guha S., Aggarwal B. B. (2009). Curcumin circumvents chemoresistance in vitro and potentiates the effect of thalidomide and bortezomib against human multiple myeloma in nude mice model. *Molecular Cancer Therapeutics*.

[8] Park J., Ayyappan V., Bae E.-K. (2008). Curcumin in combination with bortezomib synergistically induced apoptosis in human multiple myeloma U266 cells. *Molecular Oncology*.

[9] Gomez-Bougie P., Halliez M., Maïga S. (2015). Curcumin induces cell death of the main molecular myeloma subtypes, particularly the poor prognosis subgroups. *Cancer Biology & Therapy*.

[10] Bhattacharjee H., Carbrey J., Rosen B. P., Mukhopadhyay R. (2004). Drug uptake and pharmacological modulation of drug sensitivity in leukemia by AQP9. *Biochemical and Biophysical Research Communications*.

[11] Leung J., Pang A., Yuen W.-H., Kwong Y.-L., Tse E. W. C. (2007). Relationship of expression of aquaglyceroporin 9 with arsenic uptake and sensitivity in leukemia cells. *Blood*.

[12] Chau D., Ng K., Chan T. S.-Y. (2015). Azacytidine sensitizes acute myeloid leukemia cells to arsenic trioxide by up-regulating the arsenic transporter aquaglyceroporin 9. *Journal of Hematology & Oncology*.

[13] Tsukaguchi H., Shayakul C., Berger U. V. (1998). Molecular characterization of a broad selectivity neutral solute channel. *Journal of Biological Chemistry*.

[14] Liu Z., Carbrey J. M., Agre P., Rosen B. P. (2004). Arsenic trioxide uptake by human and rat aquaglyceroporins. *Biochemical and Biophysical Research Communications*.

[15] Miao Z.-F., Chang E. E., Tsai F.-Y. (2009). Increased aquaglyceroporin 9 expression disrupts arsenic resistance in human lung cancer cells. *Toxicology in Vitro*.

[16] Chou T.-C., Talalay P. (1984). Quantitative analysis of dose-effect relationships: the combined effects of multiple drugs or enzyme inhibitors. *Advances in Enzyme Regulation*.

[17] Verkman A. S. (2013). Aquaporins. *Current Biology*.

[18] Agre P., Bonhivers M., Borgnia M. J. (1998). The aquaporins, blueprints for cellular plumbing systems. *Journal of Biological Chemistry*.

[19] Lindskog C., Asplund A., Catrina A., Nielsen S., Rützler M. (2016). A systematic characterization of aquaporin-9 expression in human normal and pathological tissues. *Journal of Histochemistry and Cytochemistry*.

